# Fault Location Based on Synchronized Measurements: A Comprehensive Survey

**DOI:** 10.1155/2014/845307

**Published:** 2014-02-16

**Authors:** A. H. Al-Mohammed, M. A. Abido

**Affiliations:** ^1^Saudi Electricity Company, Dammam, Saudi Arabia; ^2^Electrical Engineering Department, King Fahd University of Petroleum & Minerals, KFUPM, Dhahran, Saudi Arabia; ^3^Electrical Engineering Department, Faculty of Engineering, Menoufia University, Shebin El-Kom, Egypt

## Abstract

This paper presents a comprehensive survey on transmission and distribution fault location algorithms that utilize synchronized measurements. Algorithms based on two-end synchronized measurements and fault location algorithms on three-terminal and multiterminal lines are reviewed. Series capacitors equipped with metal oxide varistors (MOVs), when set on a transmission line, create certain problems for line fault locators and, therefore, fault location on series-compensated lines is discussed. The paper reports the work carried out on adaptive fault location algorithms aiming at achieving better fault location accuracy. Work associated with fault location on power system networks, although limited, is also summarized. Additionally, the nonstandard high-frequency-related fault location techniques based on wavelet transform are discussed. Finally, the paper highlights the area for future research.

## 1. Introduction

Transmission and distribution lines are exposed to faults that are caused by different reasons such as short circuits, birds, and storms. Most of these faults result in mechanical damage of power lines which must be repaired before returning the line to service. Power line faults must be located accurately to allow maintenance crews to arrive at the scene and repair the faulted section as soon as possible. Rugged terrain and geographical layout cause some sections of power transmission lines to be difficult to reach. Therefore, robustness of the accurate fault location determination under a variety of power system operating constraints and fault conditions is an important requirement [1, 2].

Generally, fast and accurate fault location will expedite supply restoration and enhance the supply quality and reliability. In addition, this will minimize the customer inconvenience. Therefore, fault location can be considered as one of the first functions to be integrated into modern substation control system [2, 3].

Varieties of fault-location algorithms have been developed and presented in the literature. The majority of them are based on an impedance principle, making use of the fundamental frequency voltages and currents. Fault-location algorithms based on traveling-wave phenomenon, high-frequency components of currents and voltages generated by faults, and artificial intelligence such as fuzzy neural network [4] have also been developed. Depending on the availability of the fault-locator input signals, fault location algorithms can be categorized as one-end, two-end, and multiend. This survey is concerned with the two-end and multiend algorithms utilizing synchronized measurements. A Fault location algorithm based on aforesaid measurements proves to be robust under power swing and out-of-step conditions [5] and has been proposed [6] as part of a strategy that aimed at preventing or mitigating the cascading blackouts that involve relay misoperations or inadequate local diagnostic support.

Accuracy evaluation of various fault location algorithms, as reported in the literature, takes into account different combination of the following main factors [2]:

- fault position (location),
- fault type,
- fault resistance,
- prefault power flow and its direction,
- strength of equivalent sources behind the line terminals,
- line imbalance due to lack of transposition,
- inaccurate impedance data of the line,
- presence and status of series and shunt compensating devices with metal oxide varistors (MOVs),
- fault inception angle,
- VTs and CTs transient and steady errors.

In this survey, the issue of fault location utilizing two-end synchronized measurements is addressed in Section 2. Fault location on three-terminal and multiterminal lines is then presented in Section 3. A special attention shall be paid while developing a fault location algorithm for a series-compensated transmission line in order to locate any fault accurately. Therefore, Section 4 is devoted to discuss this matter. In order to overcome the limitations of the conventional fault location algorithms, some work has been carried out to locate power system faults in an adaptive manner and this subject is reported in Section 5. In comparison with the work pertaining to fault location on transmission lines, there is a quite limited published work related to fault location on power system networks as highlighted in Section 6. A short review of the nonstandard high-frequency-related fault location techniques based on wavelet transform is given in Section 7. Area for future research is highlighted in Section 8.

## 2. Fault Location Utilizing Two-End Synchronized Measurements

At present, phasor measurement units (PMUs) have come out of their academic infancy with commercial viability. They represent a revolution in power systems monitoring and control. A PMU can measure current and voltage and calculate the phase angle. Therefore, real-time calculation of phase angles around the system can be achieved. This can be attributed to time stamping and synchronization that are not available with traditional measurement. With the satellite GPS (global positioning system) availability, digital measurements at different line terminals can be performed synchronously. A synchronized measurement system includes the phase-angle data in addition to the magnitudes. The phase angles are measured with respect to an arbitrary but common reference. Having been known the absolute time of the measurements, phase information can be obtained. The time for all measurements must be synchronized with a time reference that must be the same for all local systems. This time reference is obtained from the GPS [7].

The potential uses of the subsecond GPS-synchronized phasor data collected from various locations within an electric power system promise endless benefits for the applications targeting reliable operation of electric power system [8]. Various PMU applications in power systems including fault location [9–15] have been reported. Merging of time correlated information from PMU, SCADA, and nonoperational data has also been suggested [16] to improve the effectiveness of alarm processing, accuracy of fault location, and ability to detect cascades.

**Figure 1:**
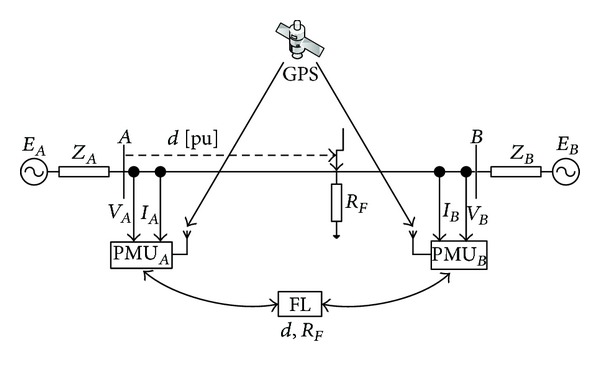
A schematic diagram for two-end synchronized fault-location arrangement using PMUs.

Two-end fault location algorithms have been proposed with the aim of overcoming the limitations of the one-end fault location techniques and improving fault-location accuracy. A schematic diagram for two-end synchronized fault-location arrangement using PMUs is shown in Figure 1 [2].

Fault location algorithms based on two-end synchronized measurements have been developed using either complete or incomplete two-end measurements. With the use of complete two-end measurements the three-phase voltages and currents are utilized. In case of incomplete two-end measurements, the following options are of interest [2]:

- three-phase voltages from both ends with three-phase current from only one end,
- three-phase currents from both ends with three-phase voltage from only one end,
- three-phase voltages from both ends.

Various fault location algorithms utilizing complete two-end synchronized measurements [1, 17–39] have been developed. Both iterative [17] and noniterative [24, 26] methods have been proposed to locate faults on a single transmission line. In the noniterative method, an analytical synchronization of the unsynchronized voltage and current measurements from two ends of a line is performed with use of the determined synchronization operator. Then, the synchronized measurements are used to calculate the distance to fault. Simultaneous usage of two kinds of symmetrical components for determining the synchronization operator makes the calculations simple and highly accurate.

Fault location taking into account the arc faults has been addressed in [21, 25, 29–32, 35]. The proposed fault detection/location technique [21, 35] for both arcing and permanent faults is achieved by a combination of a fault detection index and a fault location index which are obtained by processing synchronized fundamental phasors. In order to discriminate between arcing and permanent faults, the proposed technique estimates the amplitude of arc voltage by least error squares method through the measured synchronized harmonic phasors caused by the nonlinear arc behavior. The discrimination is then achieved by comparing the estimated amplitude of arc voltage to a given threshold value. In order to eliminate the error caused by exponentially decaying dc offset on the computations of fundamental and harmonic phasors, an extended discrete Fourier transform algorithm is also proposed. In [25, 29, 30, 32], the electric arc is considered as a source of higher harmonics and is included in the complete fault model accordingly. The developed algorithm can determine both the arc and the fault resistance. In [31], the fault location algorithm is derived in the time domain. The faulted phase voltage is modeled as a serial connection of fault resistance and arc voltage. The algorithm does not require the line zero sequence resistance as an input datum. The influences of remote infeed, fault resistance, higher order harmonics, and network topology are investigated.

For short and medium-length lines using the lumped model is usually sufficient. In order to improve fault-location accuracy, especially in the case of long-length lines, the distributed nature of overhead-line parameters has to be considered [2]. Algorithms presented in [20, 33, 37, 38] take such requirement into account by representing long lines with distributed parameters where shunt capacitance is included in the line model.

Transmission systems may sometimes consist of an overhead line in combination with an underground power cable. The fault location scheme developed in [22, 36] for such systems requires synchronized phasor measurement data at one end of the transmission line and the most far end of the power cable. The algorithm is derived using distributed line model, modal transformation theory, and discrete Fourier transform. In [39], a fault location technique for two-terminal multisection compound transmission line is presented.

The fault location algorithm presented in [34] takes the three-phase unbalance into consideration. The algorithm models the line with its distributed parameters and uses the theory of mode transformation. In [40], a fault location algorithm in joint parallel lines is proposed using six-sequence fault components in fault location. Although the algorithm is not influenced by factors such as the load current, the operating mode of the power system, or the fault resistance, the associated percentage error can reach up to 2%. A fault detection/location algorithm on transmission line based on linear state estimation is presented in [41] where fault location and voltage of fault point are added as the new state variables in a linear state estimator based on PMU data.

Fault location algorithms based on incomplete two-end synchronized measurements utilizing only three-phase voltages from both line ends [42–45] have also been developed. Algorithm proposed in [43] can be applied for both transposed and untransposed lines and algorithms presented in [44, 45] suit single or double transmission lines. However, algorithm suggested in [44] utilizes, in addition to the synchronized voltage measurements at both ends of the faulted line, the synchronized voltages at neighboring nodes. Although the method is highly accurate, the number of utilized PMUs is not optimal. Fault location algorithms utilizing only synchronized measurements of two-end voltages have the advantage of being immune to CT saturation as they completely reject the currents from the input signals.

## 3. Fault Location on Three-Terminal and Multiterminal Lines

Generally, multiterminal lines are those having three or more terminals with substantial generation behind each. Similarly, tapped lines are those having three or more terminals with substantial power generation behind at maximum two of them. Multiterminal and tapped lines are used for economical or environmental reasons. The taps feed only loads, that is, passive networks, while at the remaining terminals they are terminated by active networks [2].

Various fault location algorithms on three-terminal lines are presented in [46–52]. In [46], a fault location method is developed based on synchronized measurements of three-phase current from all three terminals and additionally three-phase voltage from the terminal at which a fault locator is installed. The delivered fault-location algorithm consists of three subroutines designated for locating faults within particular line sections and a procedure for indicating the faulted line section. An approach for fault location on EHV teed feeders [47] utilizes synchronized voltages and currents at all three ends of a teed feeder. Measurements are then digitally filtered to accurately extract the power frequency phasors. In this approach, use is made of superimposed modal components of signals so as to minimize errors arising in accuracy due to line loading or source impedances. Algorithms presented in [48, 49] use synchronized voltage and current data from two terminals only. They are not influenced by fault resistance, fault location, prefault loading conditions, source impedance, and fault types.

Algorithms discussed in [53–59] are related to multiterminal lines. The iterative method presented in [53] uses synchronized voltage and current measurements from all terminals. Current measurements, however, were avoided in [54] to overcome current-transformer errors in the current measurements that can be as high as 10%. In [56], a universal noniterative fault location technique for N-terminal transmission lines based on two-terminal fault location technique is presented. The method discussed in [57] is also noniterative and it is based on distributed line model and synchronized positive sequence voltage and current phasors. In [55], a fault location algorithm for transmission line with tapped legs is developed. The algorithm only uses the synchronized phasors measured on two terminals of the original line to calculate the fault location. The algorithm does not need the model of tapped leg and, therefore, can be applied to any type of tapped leg such as generators, loads, or combined system.

## 4. Fault Location on Series-Compensated Lines

**Figure 2:**
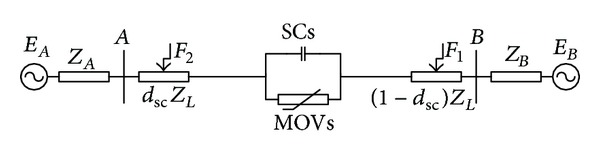
Single transmission line compensated with SCs and MOVs installed at midpoint.

**Figure 3:**
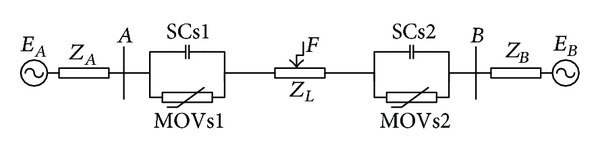
Transmission line compensated with SCs and MOVs installed at both ends.

The one-line diagrams of a series-compensated transmission line with series capacitors (SCs) and MOVs installed at midpoint and at both ends of the line are shown, respectively, in Figures 2 and 3. MOVs are installed to protect SCs against overvoltages.

Various fault location algorithms on series-compensated lines are presented in [60–65]. A fault location algorithm, presented in [60–62], does not need the series device model and information of the protection function of series device to predict the voltage drop. Instead, two iteration steps, prelocation step, and correction step are used to calculate the voltage drop and fault location. The algorithm can be applied to any series FACTS system with a very high accuracy.

Use of instantaneous values for fault location of series-compensated transmission lines while avoiding the accuracy limitation caused by the operation of MOV is discussed in [63]. The method requires only a short duration of fault measurement data to estimate the location and can be applied with minimum filtering of high frequencies. It is independent of the fault type and does not require the fault to be pure resistive. However, it requires knowledge of the source impedance at both ends of the line.

In [64], a fault location algorithm based on distributed time domain line model for a transmission line with a FACTS device connected in series is presented. The algorithm can be applied to any series FACTS compensated line since the series device model and knowledge of the operating mode of the compensating device are not utilized to compute the voltage drop across the series device during the fault period. Filtering of DC and other frequency components is not needed. The algorithm is not sensitive to fault resistance and fault inception angle and does not require knowledge of source impedance.

## 5. Adaptive Fault Location

The prime aim of adaptive fault location algorithms is to achieve a better fault location accuracy. The idea of adaptive fault location on transmission lines boils down to proper estimation of line parameters and system impedance. The environmental conditions and operation history of the transmission line affect its sag. As the conductor current increases, its temperature increases and, consequently, its sag. The line resistance changes with the line temperature. In addition, the line reactance will change since it depends on the distance between the phase conductors which is affected by the line sag. Therefore, the uncertainty of the line parameters could affect substantially the accuracy of the fault location. The effect of such uncertainty can reach up to 6-7% if the parameters vary 20% of the practical parameters. It is worth mentioning that the power utility usually provides very ideal parameters of the line that do not take into consideration its operation history [66].

With normal operation, PMU can measure the voltage at both ends of the line along with its current. The line parameters can be calculated using the prefault phase and amplitude measurements of the voltages and currents. Four methods are presented in [67] to identify transmission line impedance parameters from synchronized measurements.

Algorithms discussed in [66, 68–81] are related to adaptive fault location on transmission lines. All adaptive fault location algorithms presented in the above-mentioned references utilize synchronized voltage and current measurements at both ends of a transmission line. Adaptive fault location for aged power cables is presented in [70, 79]. The algorithm is incorporated with distributed line model, modal transformation theory, and discrete Fourier transform. It solves the problem of cable changing parameters especially the change of the relative permittivity over its age and, thus, for the operating positive, negative, and zero-sequence capacitance changes.

Algorithm presented in [71] utilizes synchronized measurements for online estimation of line parameters. A fault location index in terms of Clarke components of the synchronized voltage and current phasors is proposed to calculate the fault location. Also, a discrete Fourier transform-based algorithm is proposed to eliminate system noise and measurement errors. This work has been extended in [76, 77] by adding a fault detection index to the algorithm. A similar adaptive relaying scheme has been developed in [74, 75].

An adaptive fault protection scheme for transmission lines is discussed in [68, 69]. The work includes fault detection, direction discrimination, classification, and location. Both fault detection and fault location indices are derived by using two-terminal synchronized measurements incorporated with distributed line model and modal transformation theory. The fault detection index is composed of two complex phasors and the angle difference between the two phasors determines whether the fault is internal or external to the protected zone. The fault types can be classified by the modal fault detection index. The proposed scheme also combines online parameter estimation to assure protection scheme performance and to achieve adaptive protection. Simulation studies show that fault location accuracy is high under various system and fault conditions.

An adaptive protection scheme is presented in [72, 73] for both transposed and untransposed parallel transmission lines based on the distributed line model. The fault detection and location indices are derived using the eigenvalue/eigenvector theory to decouple the mutual coupling effects between parallel lines. The two proposed indices are used in coordination such that the internal and external fault events can be distinguished. By online estimating of line parameters under the actual system conditions, the proposed scheme responds more accurately to power system faults.

In [80], an adaptive fault location algorithm for transmission line tapped with a source of generation using the concept of superimposed voltage and current phasors is discussed. A discrimination index is proposed to identify the faulted section while taking the effects caused by tapped lines into account. The equivalent source impedance outside the considered transmission lines is estimated online.

Adaptive fault location for single, double, and teed transmission lines is addressed in [66]. In the proposed algorithm, line parameters are calculated online. In addition, suddenly changed voltage and current are utilized to obtain suddenly changed positive voltage and current components to solve the system's impedance at the fault time that exactly reflects the generation mode of the power system. The subject of adaptive fault location for double-circuit transmission lines has also been discussed in [81] where a six-sequence fault component method is employed to implement fault location. Line parameters are estimated online and the line is represented with its distributed parameters.

## 6. Fault Location in Power System Networks

Fault location in distribution networks creates new problems compared with the same task in HV and EHV transmission lines. In HV and EHV networks each transmission line may be equipped with a dedicated fault locator (FL). In such a case, the FL algorithm is a numerical procedure that converts voltage and current into a single number being a distance to fault. The distribution networks, in contrast to the transmission lines, are usually nonhomogeneous with branches and loads along the line which make the fault location difficult [2].

Various fault location algorithms in power system networks are presented in [82–99]. A method for location of single phase to ground faults in distribution network based on wide-area synchronizing information is discussed in [82]. Network lines are modeled with their distributed parameters and a fault location function is constructed according to the relations between fault current and fault distance. The transverse fault current corresponding to the hypothetic fault distance is obtained using synchronized voltage and currents from two ends of a line. The maximum transverse fault current is obtained using the interactive search algorithm on the whole line and the distance corresponding to the maximum of the transverse fault current is the fault distance.

In [84], a fault location algorithm for urban distribution system with distributed generation is presented. The method uses only currents and thus avoids the need for installation of voltage transformers. A differential current ratio (DCR) contains the differential current information to describe the feature of the faulted segment and differential current information of normal one. The faulted segment would have a DCR of a value less than one while the normal one would have a DCR greater than one. The largest fault current would be sensed by one FTU in the faulted segment.

Fault-location observability with minimum installation of PMUs in a power system network is an optimization problem that has been solved by different methods such as GA [88], branch and bound [89], and tabu search [94]. The aim here is to utilize synchronized measurements of optimally installed PMUs in a suitable algorithm to locate faults that occur anywhere in the network.

Some attempts to use discriminant analysis theory of multivariate statistical analysis theory [90], cluster analysis theory [91], power flow fingerprint [92], depth first search of graph theory [93], neural networks [86, 96], structural analysis [95], and principal component analysis (PCA) [97] have also been reported for fault location in power system networks. Fault location methods for large transmission networks are proposed in [98, 99] utilizing PMU voltage measurements.

## 7. Fault Location Using Wavelet Transform

**Figure 4:**
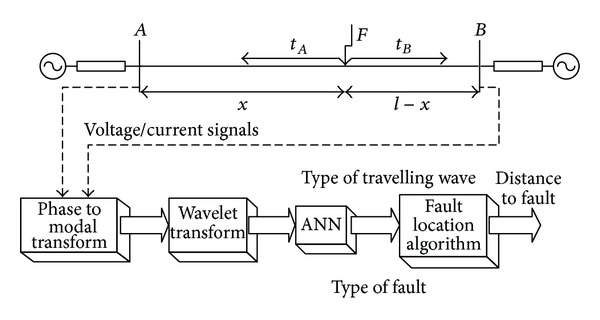
Illustration of a distorted voltage analysis using wavelet transform.

The idea of the wavelet transform- (WT-) based fault location method is illustrated in Figure 4. Measurement of arriving times of waves at the terminal buses, together with knowing the velocity of these waves, allows determination of a distance to fault. Thus, the problem in fact boils down to proper detection of arriving waves, to distinguish the type of these waves (direct waves or reflected waves) and to capture times of their arrival at terminal buses. All these tasks can be directly realized by using wavelet transforms (time location of wave) combined with ANN (distinguishing kinds of wave) [2].

Various fault location algorithms using wavelet transform are presented in [85, 100–105]. A fault-location scheme for multiend aged cable system utilizing synchronized measurements at the two terminals of each cable is presented in [101]. The developed scheme is applied on the modal coordinates instead of the phase coordinates. It can eliminate the impact of the change in the propagation velocity of the travelling waves on the fault-location calculations. This solves the problem of cable changing parameters especially the change of the relative permittivity of the cable with age.

The application of discrete wavelet transform (DWT) to fault location on power distribution lines with tapped loads is investigated in [102]. When a fault occurs, a transient wave travels from the fault point to substation busbars and load terminals. The time taken for the fault generated transient wave to arrive at busbars or load terminals depends on the distance traveled and the velocity of the travelling wave. Fault transient detectors are installed at substation busbars and load terminals to capture the time taken for the transient to arrive and be synchronized with a GPS clock. From the recorded time and the topological structure of the network, fault location is deduced.

Fault location for teed circuits with mutually coupled lines and series capacitors is proposed in [104], based on DWT, by processing of travelling waves to extract the arrival times of fault initiated waves reflected from the discontinuities. The method is not influenced by series compensation rate and its location, fault resistance, fault type, and any existing mutual coupling between the lines.

Fault location for three-terminal transmission lines is addressed in [103, 105] where WT is used to analyze high and low frequency components generated by a fault. The proposed fault location scheme is based on travelling waves and on calculation of the fundamental frequency component. The method is independent of fault impedance, fault type, fault inception angle, fault position, and mutual coupling effects.

## 8. Future Work

In case two-end synchronized measurements are utilized for fault location, it is possible to formulate more equations than the number of unknowns. The resulting redundancy may be explored to obtain certain improvements in fault location. Also, values of line parameters may not be always available in reality and, therefore, fault location techniques not requiring line parameters may be developed. Moreover, the work of designing a fault location approach capable of detecting, identifying, and removing the possible bad measurements seems interesting as it helps achieve better fault-location accuracy. Finally, application of expert systems for fault location in power systems may also be an area for future research.

## 9. Conclusion

This paper presented a comprehensive survey on fault location algorithms utilizing synchronized measurements. Algorithms based on two-end synchronized measurements have been reported and discussed. Published work related to fault location algorithms on three- and multiterminal lines has been summarized. Additionally, the paper presented the fault location algorithms on series-compensated transmission lines. Some light was shed on adaptive fault location algorithms. Fault location in power system networks and fault location based on wavelet transform have also been highlighted and discussed. Area for future research has been suggested.
